# Isoproterenol Acts as a Biased Agonist of the Alpha-1A-Adrenoceptor that Selectively Activates the MAPK/ERK Pathway

**DOI:** 10.1371/journal.pone.0115701

**Published:** 2015-01-21

**Authors:** Alicja. J. Copik, Aleksander Baldys, Khanh Nguyen, Sunil Sahdeo, Hoangdung Ho, Alan Kosaka, Paul J. Dietrich, Bill Fitch, John R. Raymond, Anthony P. D. W. Ford, Donald Button, Marcos E. Milla

**Affiliations:** 1 Biochemical Pharmacology, Inflammation Discovery, Roche Palo Alto LLC, 3401 Hillview Drive, Palo Alto, CA 94304, United States of America; 2 Discovery Technologies, Roche Palo Alto LLC, 3401 Hillview Drive, Palo Alto, CA 94304, United States of America; 3 Nephrology Division, Department of Medicine, Medical University of South Carolina, and Medical and Research Services, Ralph H Johnson Veterans Affairs Medical Center, Charleston, South Carolina 29425, United States of America; San Diego State University, UNITED STATES

## Abstract

The α_1A_-AR is thought to couple predominantly to the Gα_q_/PLC pathway and lead to phosphoinositide hydrolysis and calcium mobilization, although certain agonists acting at this receptor have been reported to trigger activation of arachidonic acid formation and MAPK pathways. For several G protein-coupled receptors (GPCRs) agonists can manifest a bias for activation of particular effector signaling output, *i.e.* not all agonists of a given GPCR generate responses through utilization of the same signaling cascade(s). Previous work with Gα_q_ coupling-defective variants of α_1A_-AR, as well as a combination of Ca^2+^ channel blockers, uncovered cross-talk between α_1A_-AR and β_2_-AR that leads to potentiation of a Gα_q_-independent signaling cascade in response to α_1A_-AR activation. We hypothesized that molecules exist that act as biased agonists to selectively activate this pathway. In this report, isoproterenol (Iso), typically viewed as β-AR-selective agonist, was examined with respect to activation of α_1A_-AR. α_1A_-AR selective antagonists were used to specifically block Iso evoked signaling in different cellular backgrounds and confirm its action at α_1A_-AR. Iso induced signaling at α_1A_-AR was further interrogated by probing steps along the Gα_q_ /PLC, Gα_s_ and MAPK/ERK pathways. In HEK-293/EBNA cells transiently transduced with α_1A_-AR, and CHO_α_1A_-AR stable cells, Iso evoked low potency ERK activity as well as Ca^2+^ mobilization that could be blocked by α_1A_-AR selective antagonists. The kinetics of Iso induced Ca^2+^ transients differed from typical Gα_q_- mediated Ca^2+^ mobilization, lacking both the fast IP_3_R mediated response and the sustained phase of Ca^2+^ re-entry. Moreover, no inositol phosphate (IP) accumulation could be detected in either cell line after stimulation with Iso, but activation was accompanied by receptor internalization. Data are presented that indicate that Iso represents a novel type of α_1A_-AR partial agonist with signaling bias toward MAPK/ERK signaling cascade that is likely independent of coupling to Gα_q_.

## Introduction

Adrenoceptors (AR) belong to the large family of G protein-coupled receptors (GPCRs), also known as seven-transmembrane receptors (7-TMRs), which transduce extracellular stimuli into cellular responses. Adrenoceptors respond to the endogenous catecholamines norepinephrine and epinephrine, and mediate critical functions of the central and peripheral nervous systems. They were initially subdivided into two main types, α- and β-, based on the rank orders of potency of norepinephrine, epinephrine and Iso as well as the physiological outcome of the response (contraction vs. relaxation) [1,2]. With the discovery of new synthetic and more selective ligands, new receptor subtypes have been identified within each of the two groups. β- AR now includes β_1_, β_2_, and β_3_- subtypes while α- is subdivided into α_1_- and α_1_- [3–6]. Introduction of molecular cloning confirmed the existence of these genetically and pharmacologically distinct subtypes of β- AR and allowed a final classification of the α_1_- subgroup into α_1A_-, α_1B_- and α_1D_- [7] and α_1_- into α_2A_-, α_2B_- and α_2C_- [8] ARs.

Iso has been one of the most commonly used agonists for differentiation of α- and β-ARs. At low concentrations (1–100 nM) Iso causes smooth muscle relaxation through its action at β-ARs, a property that prompted its introduction for the treatment of asthma, chronic bronchitis and emphysema. Even though very selective for the β-AR class, several groups reported that Iso, at high doses (4 μM and higher) also evoked α- mediated responses leading to the contraction of smooth muscles of rabbit aorta and posterior vena cava as well as of rat vas deferens [9–14]. High doses of Iso were also shown to increase blood pressure in rabbits [1], and cause arterial hypertension in anesthetized cats and dogs [15,16]. The involvement of α-AR in mediating the physiological effects of Iso was implicated in these and other studies by the ability of antagonists dibenamine, phenoxybenzamine or phentolamine to block responses [11,14,15].

More recently, observations of Ca^2+^ mobilization responses in rat parotid acinar cells in response to high concentrations of Iso (1–200 μM) led to a long running debate of how Ca^2+^ is involved in cAMP-mediated amylase release, and whether this response is mediated solely by β-AR [17,18]. Subsequent studies in rat parotid acinar cell preparations revealed prazosin sensitivity for the Iso-mediated Ca^2+^ mobilization response, indicating Iso activation of α-AR [19,20] although the subtype involved was not identified. Thus, although compelling historical precedents exist for Iso agonism at α_1_-ARs, no studies focused on the signaling mechanisms or α_1_-AR receptor sub-types involved. The use of Iso in basic and clinical studies would clearly benefit from greater mechanistic understanding of Iso- mediated signaling via α-ARs.

Iso binds with relatively high affinity to all three β-AR subtypes (*K*_i_: 0.22 μM at β_1_-, 0.46 μM at β_2_- and 1.6 μM at β_3_-AR in presence of GTP; 0.02 μM at β_2_-AR in its absence [21,22]), acting as a high intrinsic efficacy (full) agonist. Thus, Iso-bound β-AR couples to Gα_S_ leading to stimulation of adenylyl cyclase, cAMP production, and phosphorylation of protein kinase A (PKA). In addition to the activation of this “canonical” pathway, Iso is highly efficacious at inducing β_2_-AR mediated signaling via G-protein coupled receptor kinases-(GRK) and β-arrestin. This leads to receptor phosphorylation, recruitment of c-Src and activation of MAPK signaling pathways among others (reviewed in [23].

It has also been shown that Iso at higher doses (above 100 nM) induces G protein-independent signaling at β_2_-ARs [24]. In mouse embryonic fibroblasts (MEFs), stimulation of β_2_-AR by Iso results in a biphasic concentration–dependent increase in extracellular signal-regulated protein kinase (ERK) activity. The high potency phase was found to be dependent on Gα_S_ while the low potency phase was not. Sun *et al.* combined the use of MEF cells from various knock-out mice with biophysical studies testing the interaction of the purified components (β-AR and Src) to show that the low potency phase reflects direct interaction and activation of Src by the Iso-activated β_2_-AR. The high concentrations of Iso used in this and several other studies [18,25,26] may also lead to activation of α-ARs with signaling consequences that are not well-characterized at the molecular level. Thus, there is a great need to explore the mechanism of Iso signaling through α-ARs.

We investigated the Iso initiated signaling in transiently transduced HEK-293/EBNA cells expressing quasi-physiological levels of α_1A_-AR. Since HEK-293 cells endogenously express β_2_-AR, this system gave us the ability to monitor Iso activation of several cellular events in the presence and absence of α_1A_-AR [27]. In untransduced cells, we observed Iso- induced monophasic cAMP and ERK activation as well as Ca^2+^ mobilization with efficacy within the range expected for this agonist acting at β_2_-AR. In contrast, in cells transiently transduced with α_1A_-AR, Iso evoked biphasic concentration-dependent activation of ERK activity as well as Ca^2+^ mobilization. The high potency phase of the concentration-effect relation was sensitive to a β_2_- selective antagonist, while the low potency phase was blocked by application of α_1A_-AR-selective antagonists. Iso was found to be an agonist at recombinantly expressed α_1A_-AR subtype in a manner which recruits signaling mechanisms distinct from those seen with NE or selective α_1A_-AR agonists. Our data suggest that Iso induces a α_1A_-AR-mediated signaling mode biased toward MAPK/ERK and likely independent Gα_q_. We also show evidence indicating that this signaling mode involves receptor internalization.

## Materials and Methods

### Materials and reagents

Reagents were purchased from the following commercial suppliers: A-61603, xamoterol, procaterol and fenoterol from Tocris (Ellisville, MO); norepinephrine, prazosin, phentolamine, ICI 118551, atenolol, salbutamol, propranolol, probenecid, BSA and glucose from Sigma-Aldrich (St. Louis, MO); isoproterenol and oxymetazoline from MP Biomedicals (Irvine, CA), sodium butyrate from Alfa Aesar (Ward Hill, MA); HEPES, HBSS, 3-isobutyl-1-methylxanthine (IBMX) from Axxora (San Diego, CA) and Fluo3-AM from Invitrogen (Carlsbad, CA). [^3^H]-Prazosin and [^125^I]-CYP were purchased from Perkin Elmer (Boston, MA). Crude membranes were prepared from transduced or transfected HEK-293/EBNA cells as published [28] with one modification. Cells were resuspended in the lysis buffer without sucrose and broken using a Polytron homogenizer (3 × 30 s pulses).

### Cell culture and transient transductions

Cell-based experiments were performed using suspension-adapted HEK-293/EBNA cells [27], HEK293 or CHO cells. HEK-293/EBNA cells were grown in Free Style 293 serum free medium (Invitrogen) and maintained in an incubator at 37°C, 7% CO_2_ atmosphere with constant shaking at 150 rpm. Prior to experiments, cells were transduced with baculovirus strains designed for transient expression of α_1A_-AR or aldehyde oxidase (negative control). This was performed by incubating cells with virus (MOI 100–150) for 3 to 4 h, followed by exchange into fresh serum free growth medium supplemented with 4 mM sodium butyrate (NaBu). Cells were grown for another 14 to 18 h and then examined for agonist-evoked responses in transient Ca^2+^ release, IP accumulation or pERK activation assays. The surface receptor expression density was determined by flow cytometry via immunofluorescence labeling of an N-terminal HA epitope tag on the receptor and radioligand binding in partially purified membranes.

HEK293 cells were grown in Eagle’s minimum essential medium (MEM) supplemented with 10% (v/v) heat-inactivated fetal bovine serum (Invitrogen Gibco, Carlsbad, CA) at 37°C in a humidified atmosphere of 95% air and 5% CO_2_.

### Ca^2+^ transient response assay

Cells were resuspended in Hank’s balanced salt solution (Invitrogen) supplemented with 2 mM CaCl_2_, 10 mM HEPES pH 7.4, 2.5 mM probenecid, plus 1g/L each of glucose and bovine serum albumin (BSA), and seeded in poly-D-lysine coated 96-well black plates with transparent bottom (Costar) at a density of 50,000 cells/0.1 mL per well. Cells were then incubated for 1 h at 37°C with an additional 0.1 mL of buffer containing 4 μM fluo3-AM that was diluted from a stock solution containing 1 mM fluo3-AM dye in DMSO with 10% pluronic acid. After dye loading incubation, plates were washed twice with 100 μL of buffer and refilled with 100 μL of assay buffer. In experiments testing the effect of antagonists, 25 μL of buffer from wells of washed cells were replaced with 25 μL of vehicle or 4× antagonist solution. Pre-incubation of ligand with dye-loaded cells proceeded for 5–30 minutes immediately prior to measurements of agonist-evoked responses. Agonist-evoked Ca^2+^ mobilization responses of cell populations were monitored at room temperature simultaneously in all wells of the assay plate using the plate-imaging fluorometric reader FLIPR (MDS Analytical Technologies, Sunnyvale, CA). Measurements consisted of recording baseline fluorescence signal for 10 seconds followed by addition of the test substance and 1–2 minute readings of Ca^2+^ transient responses as reported by changes in Ca^2+^ dye fluorescence (excitation 488 nm, emission 510–570 nm). The amplitudes of Ca^2+^ transient responses are reported as ΔF/F_0_, or fold-change in Ca^2+^ dye fluorescence relative to the baseline signal ΔF/F_0_ = (F-F_0_)/F_0_ +1 (where ΔF is maximal fluorescence intensity observed following agonist application, F_0_ = baseline fluorescence, or average intensity measured over the 10 s interval prior to agonist application). Image acquisition rates were varied from 1 Hz during the first 100 s of measurements to 0.5 Hz for the remaining time of the recording.

### IP and cAMP Accumulation Assays

Virally transduced HEK-293/EBNA cells were washed by centrifugation at 150 × g for 8 min, before resuspension in assay buffer (20 mM HEPES, 10 mM glucose, 1.8 mM CaCl_2_, 0.5 mM MgSO_4_, in HBSS 1X buffer, 50 mM LiCl) at 10^8^ cells/mL. Cells were placed in 384-well black polystyrene plates (Costar 3912) at 10 μL/well, and incubated with 10 μL of antagonist or vehicle at room temperature. After 10–20 minutes, 10 μL of agonist was added followed by incubation for 5 to 30 minutes. Stimulation was stopped by addition of lysis buffer. Second messenger levels were determined using a homogeneous immunoassay method with time-resolved FRET detection (IP-One HTRF, Cisbio International) according to protocols provided by the supplier. Sample fluorescence was measured with a Nanoscan plate reader (IOM, Berlin). Each data point represents the average of quadruplicate determinations and each experiment was performed at least two times independently.

### Detection of IP_1_, IP_2_ and IP_3_ inositol phosphates

Virally transduced HEK-293/EBNA cells were resuspended in growth medium and seeded in poly-D-lysine coated 96-well black plates at a density of 100,000 cells/0.1 mL per well, Cells were allowed to attach for 2 hours at 37°C in a humidified atmosphere of 95% air and 5% CO_2_. After that, the medium was replaced with agonist solution in assay buffer (20 mM HEPES, 10 mM glucose, 1.8 mM CaCl_2_, 0.5 mM MgSO_4_, in HBSS 1X buffer, 50 mM LiCl) and cells were incubated at 37°C for 30 min. Stimulation was stopped by exchange of assay buffer with lysis buffer (100 μL 0.1N HCl/MeOH), and lysis proceeded for 30 min at 4°C. Cellular levels of inositol phosphates were determined using a Thermo LC-MS detection system following a published protocol [29]. Chromatography was performed employing a BioBasic column AX (50 × 2.1 mm, Thermo). The mobile phase consisted of solvent A: 10 mM ammonium acetate, pH 6 in 30/70 acetonitrile/water, and solvent B: 1 mM ammonium acetate, pH 11 in 30/70 acetonitrile/water. A gradient solvent system was used starting with 0% solvent B, and analytes were eluted by increasing solvent B to 100% over 3 min. Each data point represents the average of triplicate determinations and each experiment was performed at least three times independently.

### MAPK activation assays

MAP kinase activation was monitored using the bead proximity-based AlphaScreen assay to detect phosphorylated ERK (*SureFire* p-ERK, PerkinElmer, Boston, MA). Virally transduced HEK-293/EBNA cells were seeded in 96 well plates with serum-free growth medium at 50,000 cells/well, followed by incubation for six hours at 37°C in 7% CO_2_. Cells were then stimulated with vehicle or agonist for 5 min at 37°C in 7% CO_2_. Agonist response was terminated following rapid removal of the medium by adding 50 μL per well of SureFire lysis buffer, followed by incubation of cells for 10 min at room temperature. Plates were stored at −80 C prior to analysis for p-ERK levels. Processing of cells for phospho-ERK detection was performed using an AlphaScreen *SureFire* p-ERK assay kit (PerkinElmer, Boston, MA) according to specifications from the manufacturer. Briefly, 10 μL of lysate were transferred to 384 well plates (OptiPlate) and combined with 17 μL of SureFire buffer containing AlphaScreen beads. Plates were incubated for 2 h at room temperature and the fluorescence signal was recorded using a Fusion plate reader (PerkinElmer), adjusted to standard AlphaScreen settings.

### Radioligand binding studies

Ligand binding was monitored using membranes prepared from NaBu-treated untransduced HEK-293/EBNA cells or cells virally transduced with the α_1A_-AR construct. [^3^H]-prazosin and [^125^I]-CYP were used as radioligands and 100 μM phentolamine or 10 μM propranolol as respective non-radioactive competitors to determine non-specific binding. For competition binding assays, unlabeled ligands (A-61603, NE, and Iso) were used to compete [^3^H]-prazosin or [^125^I]-CYP binding. Reactions were set up as described previously [27]. Affinity (p*K*_i_) values were calculated from IC_50_ values using the Cheng-Prusoff correction [30].

### Immunofluorescence staining and confocal microscopy

HEK293 cells were transiently co-transfected with FLAG-tagged α_1A_ – AR and one of the following GFP-tagged proteins: Rab5 (Rab5 Q79L) variant, Rab11 (Rab11 S25N) variant, β-arrestin-1 or β-arrestin-2. Rab5 and Rab11 were kindly provided by Dr. Marino Zerial (Max Planck Institute of Molecular Cell Biology and Genetics, Dresden, Germany). Following serum-deprivation for 24h, cells were stimulated with 1 μM A-61603 or 1 mM ISO for 2h. After treatment, cells were fixed with freshly prepared 3.7% paraformaldehyde in PBS for 15 min at room temperature. Subsequently, cells were permeabilized with 0.1% Triton X-100 (Sigma-Aldrich) in PBS for 5 min, followed by nonspecific binding site blocking with 3% normal serum (Santa Cruz Biotechnology) in PBS for 1 h. Incubation with Alexa Fluor-568 conjugated anti-FLAG antibodies in blocking solution was done as directed by the manufacturer. Confocal microscopy was performed using a Zeiss LSM 510 META laser scanning microscope (Carl Zeiss, Inc., Thornwood, NY) equipped with a 60X objective, using the following laser wavelengths: excitation at 488 nm and emission at 505–530 nm; excitation at 543 nm and emission at 560–615 nm.

### Reversible biotinylation of Cell Surface Proteins

HEK293 cells transiently transfected with α_1A_ – AR were serum deprived for 24h prior to treatments. Cells were washed with ice-cold PBS and incubated with 0.5 mg/mL of cell-impermeable sulfo-NHS-biotin (Pierce) for 30 min at 4°C to label surface proteins, followed by washing with 15 mM glycine to quench excess, unreacted biotin. Cells were further washed with PBS and incubated in serum-free medium at 37°C for 1 h, then treated with vehicle, 1 μM A-61603 or 1 mM ISO for 5, 30, or 60 min. After treatment, cells were rinsed briefly with ice-cold PBS, and either collected following stripping of cell surface biotin (intracellular receptors), or collected without biotin stripping (total cell surface and intracellular receptors). Stripping of cell surface biotinylated receptors was performed at 4°C by washing cells three times for 5 min each with ice-cold GSH cleavage buffer (50 mM GSH, 75 mM NaCl, 1mM EDTA, 1% BSA, 0.075 N NaOH). Cellular proteins were extracted with Triton lysis buffer [50 mM Tris-HCl (pH 7.4), 150 mM NaCl, 1% Triton X-100, and 5 mM EDTA] supplemented with protease inhibitor cocktail III (EMD Calbiochem, San Diego, CA), 1 mM PMSF, and phosphatase inhibitors (HALT phosphatase inhibitor cocktail, Pierce). Equal amounts of proteins (0.5 mg) were precleared by incubation for 30 min at 4°C with 30 μL of protein A/G Agarose beads (Santa Cruz Biotechnology). After brief centrifugation, supernatants were removed and incubated overnight at 4°C with 50 μL of streptavidin-agarose beads (Novagen, Madison, WI). Samples were then centrifuged and washed three times with 1 mL of Triton lysis buffer. Proteins were eluted from the beads using Laemmli sample buffer, followed by analysis via SDS-PAGE and Western blotting.

### Data Analysis

Experiments were carried out in independent replicates (n indicated in figure legends). Graphs shown reflect either pooled or representative data (see figure legends). For concentration-response analysis, results from Ca^2+^ mobilization experiments were plotted as ΔF/F_0_ vs. agonist concentration and fit to a sigmoidal concentration-response equation using the GraphPad Prism software package. In cases where biphasic concentration-response curves were observed data were fit to a two-site concentration-response model:

$$\text{F}\left(X\right)={\text{R}}_{\text{0}}\text{+}\left({\text{R}}_{\text{max}}{-\text{R}}_{\text{0}}\right){[\text{Fr}}_{1}/(1+10^{\text{X-logEC}}50,1)+\left(1-{\text{Fr}}_{1}\right)/(1+10^{\text{X-logEC}}50,2)]$$ (1),

where X is the logarithm of the agonist concentration, R_0_ and R_max_ are respectively the response minimum and maximum; Fr_1_ is the response fraction attributable to receptor characterized by half effective concentration of the first response phase (EC_50,1_), with the reminder response attributable to the second half effective concentration (EC_50_,_2_). For radioligand binding experiments, *B*_max_ values were calculated from maximum binding using the equation *B*_max_ = (SB × (IC_50_ + [L])/[L]), where SB is the specific binding expressed as fmol per mg of membrane protein, and [L] is the ligand concentration.

## Results

### Iso induces biphasic concentration-response behavior in Ca^2+^ mobilization in α_1A_- AR transduced HEK-293/EBNA cells

**Figure 1:**
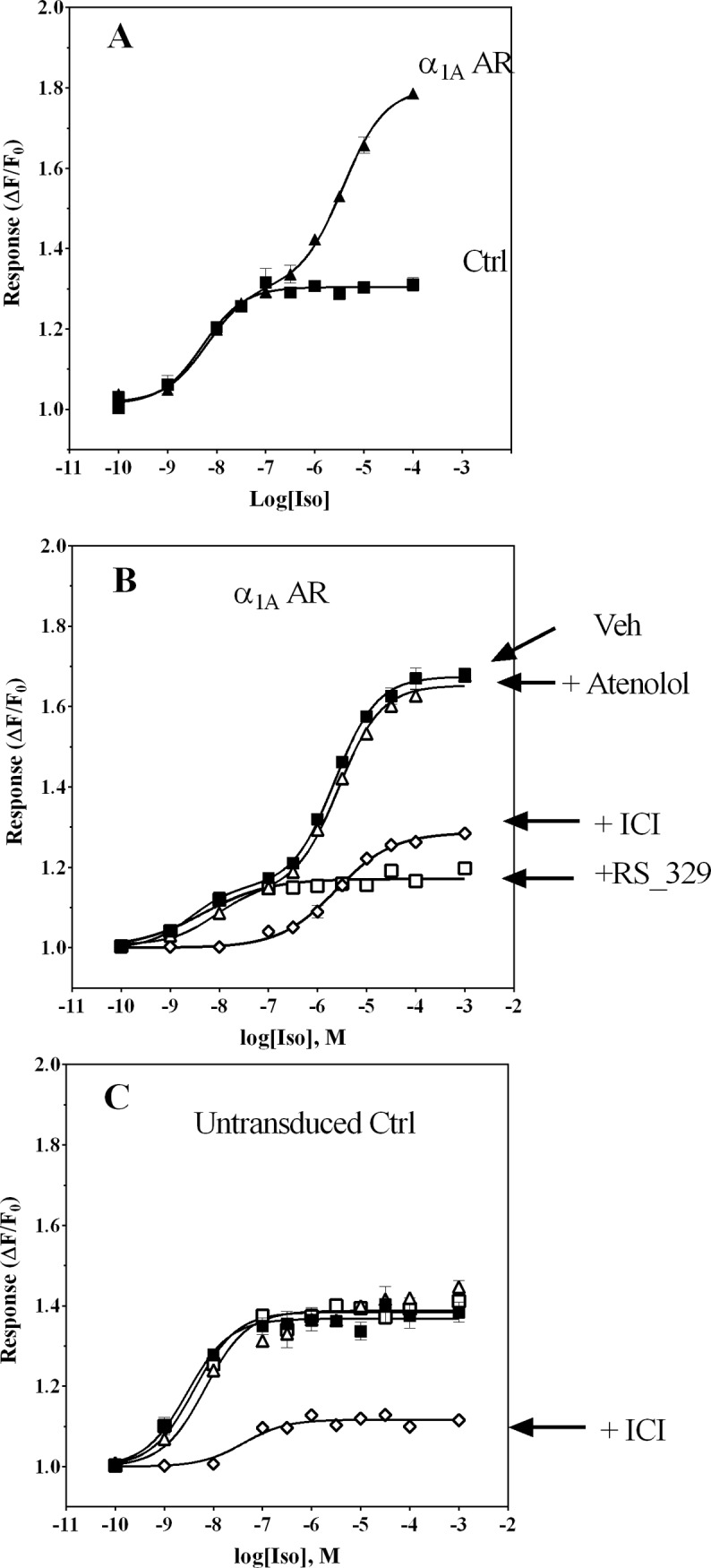
Biphasic concentration-response relationship for Iso-mediated Ca^2+^ responses in α_1A_-AR transduced HEK-293/EBNA cells. Ca^2+^ transient responses (expressed as ΔF/F_0_) were measured as a function of Iso concentration in fluo3-loaded cells by fluorometric plate imaging (FLIPR). HEK-293/EBNA cells were first exposed for 3–4 h to baculovirus encoding α_1A_-AR or an irrelevant negative control protein (aldehyde oxidase), then cultured in fresh medium containing 4 mM NaBu for 18 h prior to use in experiments as described in Materials and Methods. **Panel A**: Control cell (■) Ca^2+^ transient responses exhibited a monophasic increase in amplitude, whereas responses in cells transiently expressing α_1A_-AR (▲) revealed a biphasic concentration-response relationship, with large response amplitude at high Iso concentrations. **Panel B:** Blockade of Iso responses by pre-treatment of cells with antagonists selective for β-ARs or α_1A_-ARs reveals roles for both receptors in mediating Ca^2+^ transient responses in HEK-293/EBNA cells transiently expressing α_1A_-AR. Although the β_2_-AR-selective antagonist ICI 118551 at 10 nM (◇) abolished responses to low Iso concentrations, responses to high Iso concentrations were attenuated by only ∼ 50%. The α_1A_-AR-selective antagonist RS100329 (□) blocked only responses to high Iso concentrations. Neither phase of the concentration-response profile was affected by the β_1_-AR-selective antagonist atenolol (1 μM, Δ). Vehicle-treated cell responses represented by ■. **Panel C:** In non-transduced control cells, only the β_2_-AR-selective antagonist ICI 118551 at 10 nM (◇) was effective at blocking responses to Iso: both α_1A_-AR-selective RS100329 (10 nM, □) and β_1_-AR-selective atenolol (1 μM, Δ) were ineffective. Each data point represents an average of duplicate determinations; results shown are representative of experiments repeated at least 3 times.

In baculovirus-transduced HEK-293/EBNA cells homogenously expressing low levels of α_1A_-AR close to the ones observed in primary cells (∼400 fmol/mg protein, [27]), the β-AR selective agonist Iso evoked transient responses in Ca^2+^ mobilization. A plot of the peak amplitude of the Ca^2+^ transient, *i.e.* maximal ΔF/F_0_, as a function of agonist concentration yielded a biphasic concentration-response curve (**Fig. 1A**). This suggested the presence of two distinct mechanisms mediating the observed Ca^2+^ response. The half-maximal amplitude of the high potency phase was observed at 4.0 nM Iso, whereas the half-maximal amplitude of the lower potency phase occurred at 2.6 μM Iso (**Table 1**). Since HEK-293/EBNA cells are known to express β_2_-AR endogenously (∼100–300 fmol/mg protein [27,31]), we examined if the parental cells responded to Iso in a similar fashion. Application of Iso to HEK-293/EBNA cells transduced with negative control vector (aldehyde oxidase) resulted in monophasic concentration-dependent Ca^2+^ mobilization with EC_50_ = 4 ± 1 nM (**Fig. 1A, Table 1**), equivalent to the EC_50_ determined for the high potency phase of the biphasic dose-response curve in α_1A_-AR_HEK-293/EBNA cells. Furthermore, the maximal response (ΔF/F_0_ = 1.3) matched that of the first, high potency phase observed in α_1A_-AR-expressing cells, derived from fitting data to a biphasic concentration-response equation (ΔF/F_0_ = 1.3). This finding indicates that Iso occupancy of endogenous β_2_-AR leads to a small magnitude Ca^2+^ transient response in untransduced HEK-293/EBNA cells and potentially in the α_1A_-AR-transduced ones. Since the Iso EC_50_ in non-transduced cells corresponds to the higher potency phase of the biphasic Iso response observed in α_1A_-AR_HEK-293/EBNA cells, we used receptor-selective antagonists to further characterize the observed Ca^2+^ response. The lower potency phase of response to Iso was sensitive to the highly selective α_1A_-AR antagonist, RS100329 (p*K*_B_ = 9.6 for α_1A_-AR) (10 nM, **Fig. 1B**) [32]. The magnitude of the Iso response in presence of RS100329 (ΔF/F_0_ = 1.2) matched the magnitude of the high potency response phase observed in vehicle pretreated cells, as determined using the two-site model (ΔF/F_0_ = 1.2). Consistent with this, the potency of Iso in the presence of RS100329 (EC_50_ = 4.6 nM) corresponded well to the potency calculated for the high potency phase of biphasic dose-response curves in cells pretreated with vehicle (EC_50_ = 4.0 nM, **Table 1**). This finding indicates virtually complete blocking of the low potency phase by RS100329 with no effect on the high potency phase. On the other hand, pretreatment of α_1A_-AR_HEK-293/EBNA cells with the β_2_-AR selective antagonist ICI 118,551 (10 nM) resulted in monophasic concentration-response to Iso (**Fig. 1B**). The calculated potency of Iso in the presence of this β_2_-AR selective antagonist (EC_50_ = 2.4 μM) matched the EC_50_ of 2.6 μM determined for the low potency phase of the bipasic response to Iso in vehicle-treated cells. Interestingly, the maximal response amplitude for Iso in the presence of ICI 118,551 (ΔF/F_0_ = 1.3, estimated using a monophasic concentration-response model) was lower than the amplitude of the lower potency phase observed in vehicle treated cells (ΔF/F_0_ ∼ 1.5, estimated using a biphasic concentration-response model). This suggests some level of synergy when Iso simultaneously occupies α_1A_-AR and β_2_-AR. The β_1_-AR selective antagonist atenolol (1 μM) had no effect on the Iso concentration-response relationship (**Fig. 1B**). In untransduced HEK-293/EBNA cells, response to Iso was blocked by 10 nM ICI118551, a β_2_-selective antagonist, but not by the α_1A_-AR-selective antagonist RS100329 (**Fig. 1C**). Thus, the biphasic Ca^2+^ transient concentration-response relationship observed with Iso seems to require co-expression of both the α_1A_- and β_2_-adrenoceptors.

**Table 1:** Pharmacological parameters determined from IP and cAMP accumulation, Ca^2+^ mobilization and p-ERK1/2 assays performed with transiently transduced HEK-293/EBNA cells.

**Cell line**	**Agonist**		***K*_i_ (μM)**	**EC_50_ IP (μM)**	**EC_50_ Ca^2+^ (μM)**	**EC_50_ cAMP (μM)**	**EC_50_ p-ERK (μM)**
α_1A_-AR HEK293/EBNA	NE		^1^3.1	^1^0.90 ± 0.20	^1^0.070 ± 0.010	^2^0.67 ± 0.04	0.09 ± 0.04
	A-61603		^1^0.13	^1^0.040 ± 0.020	^1^0.003 ± 0.001	^2^0.019 ± 0.005	0.0008 ± 0.0003
	Iso	1	^5^0.44	^3^BQL	^4^0.004 ± 0.002	0.021 ± 0.020	^4^0.005 ± 0.004
		2	93	BQL	^4^2.6 ± 2.0	BQL	^4^6.0 ± 2.0
HEK293/EBNA	NE		^1a^34	BQL	^1^0.50 ± 0.10	4.9 ± 0.04	^6^ND
	A-61603		^1a^24	BQL	^1^0.003 ± 0.001	BQL	BQL
	Iso		^5^0.45	BQL	0.004 ± 0.002	0.026 ± 0.020	0.001 ± 0.001

Data were fitted to a sigmoidal dose-response or two-site concentration-response model using the GraphPad Prism software package to determine EC_50_ values, expressed as mean ± standard error.

^1^[27]

^1a^ [^125^I-CYP] used as radioligand

^2^Values determined in assays performed with HEK-293/EBNA cells not treated with NaBu and transduced with baculovirus construct yielding high pmol level expression of α_1A_-AR

^3^BQL: Below quantifiable levels

^4^ *EC*_50_ values for the Ca^2+^ mobilization and p-ERK assays were determined from fitting data to a biphasic concentration-effect model (E_q_ 1, *Materials and methods* section).

^5^ *K*_i_ of Iso at β_2_-AR and α_1A_-AR were determined as described in Materials and Methods by radioligand competition binding methods using [^125^I]CYP and [^3^H]-Prazosin, respectively, as the radioligands.

^6^ND –not determined

### Iso induces Ca^2+^-mobilization in α_1A_-AR transduced HEK-293/EBNA cells with atypical, slower kinetics

**Figure 2:**
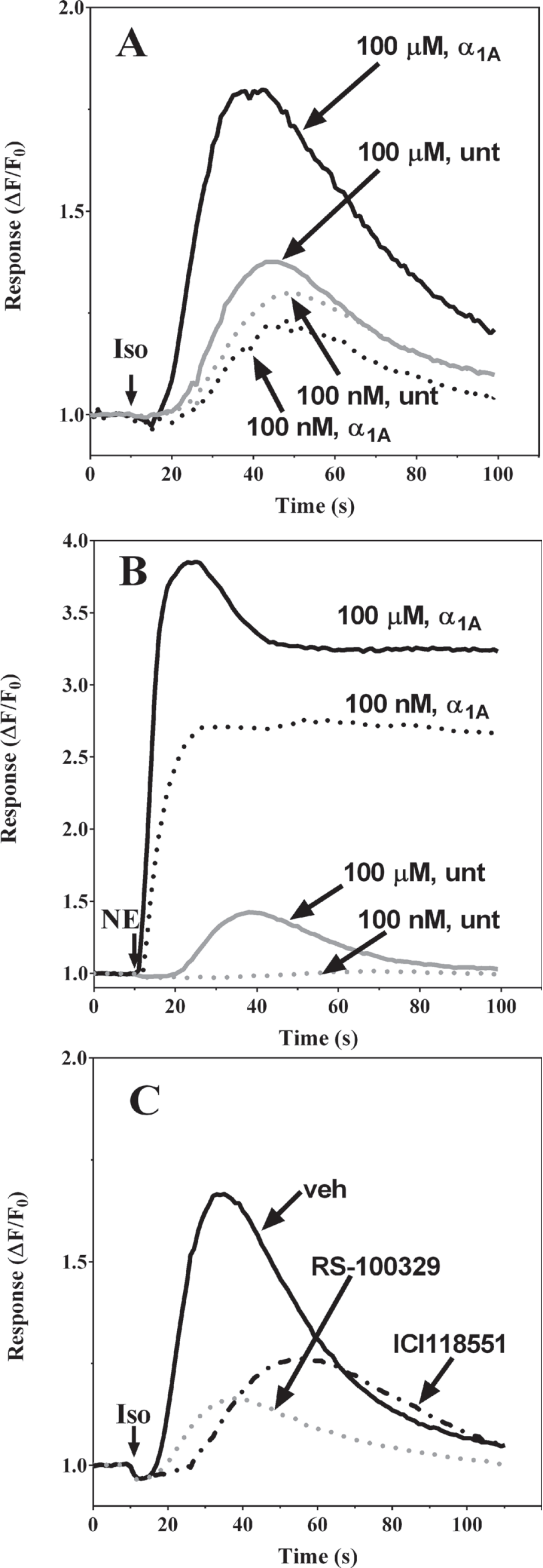
The Iso-induced Ca^2+^ mobilization response in α_1A_-AR transduced HEK-293/EBNA cells is slower in onset and shorter in duration than the response to NE. Ca^2+^ transient response kinetics (expressed as ΔF_t_/F_0_) are shown for fluo3-loaded cells monitored fluorometrically following addition of Iso or NE. HEK-293/EBNA cells were first exposed to recombinant baculoviral strains (3–4 h) encoding either α_1A_-AR or an irrelevant negative control protein (aldehyde oxidase), then cultured in fresh culture medium containing 4 mM NaBu for 18 h prior to use in experiments as described in Materials and Methods. **Panel A**: Slow onset of the Iso agonist response which returns to baseline within a 2 min interval is evident in representative traces from α_1A_-AR transduced HEK-293/EBNA cells (black lines) and negative control cells (gray lines) during responses elicited by addition of Iso (↓) at 100 nM (dashed lines) or 100 μM (solid lines). **Panel B**: Representative Ca^2+^ transients in α_1A_-AR transduced HEK-293/EBNA cells showing rapid onset and sustained NE response following application of NE (↓) at 100 nM (dashed lines) or 100 μM (solid lines) to α_1A_-AR transduced (black lines) or negative control-transduced cells (gray lines). **Panel C**: α_1A_-AR transduced HEK-293/EBNA cells exhibit distinct kinetics for Iso-mediated responses via occupancy of β-AR vs α_1A_-AR transduced, as revealed by monitoring of responses following pre-treatment of cells for 20 min with vehicle (solid black line), 10 nM RS-100329 (dashed gray line) or 10 nM ICI118551 (dashed black line). Representative traces are shown for responses to 100 μM Iso (↓).

It has been shown previously that the kinetics of intracellular Ca^2+^ accumulation mediated by α *vs*. β_2_-AR are very different, due to the distinct sources of Ca^2+^ involved with each pathway downstream from the receptor. The Gα_q_-mediated α_1_-AR transient is very fast in onset relative to the Gα_s_-mediated β_2_-AR transient [31,33–35]. To further dissect Iso-mediated contributions to observed transients, we analyzed the kinetics of Ca^2+^ responses to Iso and norepinephrine (NE) in α_1A_-AR_HEK-293/EBNA transduced and untransduced cells (**Fig. 2**). Stimulation of α_1A_-AR_HEK-293/EBNA cells with 100 μM Iso (a concentration that produces high agonist occupancy at both α_1A_-AR and β_2_-ARs) elicited a slow onset Ca^2+^ transient response (5–10 second delay from addition of agonist to the rise in Ca^2+^) that return towards baseline (i.e. decline from peak amplitude >75%) within 100 sec and lacked the sustained phase (**Fig. 2A**, solid black line). A smaller magnitude response with similar slow onset and no sustained elevated phase was observed in response to 100 nM Iso, a concentration that would achieve high fractional occupancy of β_2_-AR, yet insignificant at α_1A_-AR (**Fig. 2A**, dashed black line). Responses to application of 100 nM or 100 μM Iso in untransduced negative control cells are shown by the gray dashed and solid traces, respectively. By contrast, treatment of α_1A_-AR_HEK-293/EBNA transduced cells with 100 nM NE resulted in an almost immediate Ca^2+^ transient onset, with peak response amplitude for the population average attained within 10 seconds post-agonist addition (**Fig. 2B**, dashed black line). This response was characterized by a sustained plateau phase of elevated cytosolic Ca^2+^, not found in untransduced control cells (**Fig. 2B**, black lines vs. solid gray trace). Because NE possesses ∼ 10-fold higher affinity at α_1A_-AR relative to β_2_-AR (*K*_i_ = 3.1 μM at α_1A_-AR *vs*. 34 μM at β_2_-AR, **Table 1**), the observed response to 100 nM NE is likely driven mostly through occupancy at α_1A_-AR (**Fig. 2B**, dashed black line). Thus, the rapid and sustained Ca^2+^ elevation in response to 100 nM NE reveals an ability of this agonist to elicit Ca^2+^ transient responses through a mechanism distinct from the slow onset and transient mobilization response induced by Iso. Responses to 100 μM Iso in cells that were preincubated with the α_1A_-AR selective antagonist RS100329 or the β_2_-AR-selective antagonist ICI 118551 revealed the functional contributions of both receptors (**Fig. 2C**; gray dashed and black dashed lines, respectively): both antagonists significantly diminished responses and delayed their onset when compared to the vehicle control (**Fig. 2C**; solid black line). Interestingly, the biggest delay was observed in cells pretreated with the β_2_-AR-selective antagonist ICI 118551, a condition that isolates the α_1A_-AR component of Iso-mediated Ca^2+^ transients (**Fig. 2C**; black, dashed line).

### Effect of extracellular Ca^2+^ removal on the β_2_- and α_1A_-AR-dependent Ca^2+^ mobilization response to Iso stimulation

**Figure 3:**
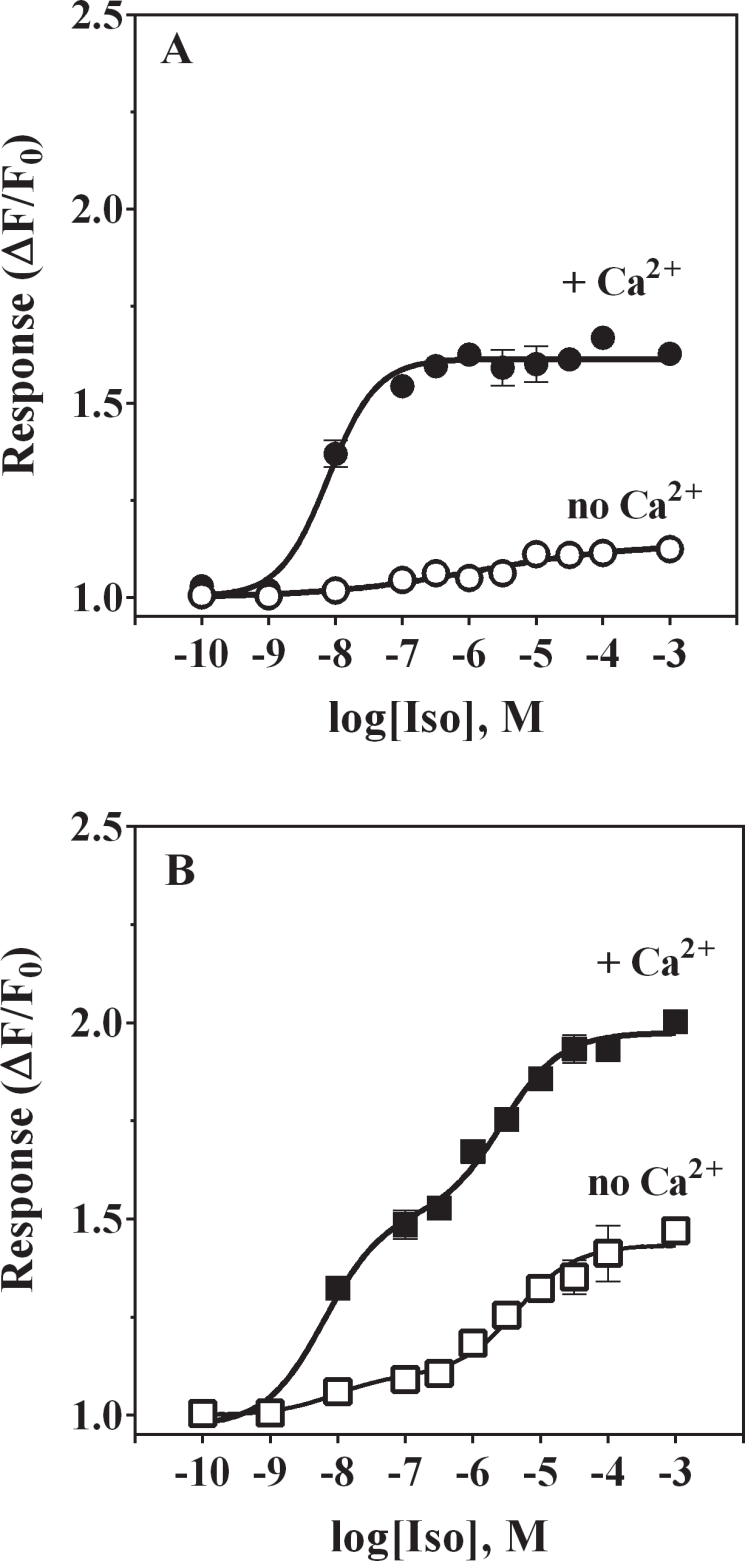
Iso-induced Ca^2+^ mobilization in α_1A_-AR transduced HEK-293/EBNA cells is partially dependent on the presence of extracellular Ca^2+^. Ca^2+^ transient responses (expressed as ΔF/F_0_) were measured as a function of Iso concentration in untransduced control cells (**Panel A**) or α_1A_-AR transduced HEK-293/EBNA cells (**Panel B**), either in the presence (filled symbols) or absence (open symbols) of 2 mM Ca^2+^ in the assay buffer. Responses in untransduced cells were virtually abolished in the absence of extracellular Ca^2+^ (**Panel A**). In α_1A_-AR transduced cells, responses to low concentrations of Iso in the absence of extracellular Ca^2+^ were also essentially abolished whereas the low potency phase of response was diminished by approximately 50% (**Panel B**). Each data point is an average of duplicate determinations; this experiment was repeated twice.

We next addressed whether observed rises in cytosolic Ca^2+^ were due to influx of extracellular Ca^2+^ by measuring Ca^2+^ mobilization in HEK-293/EBNA cells upon exposure to Iso in the presence or absence of external Ca^2+^. Omitting Ca^2+^ from the extracellular buffer eliminated Iso-evoked Ca^2+^ responses in untransduced HEK-293/EBNA cells (**Fig. 3A**). In α_1A_-AR_ HEK-293/EBNA cells, Iso-mediated responses were greatly reduced in amplitude in the absence of extracellular Ca^2+^ (**Fig. 3B**). However, the low potency phase of the concentration-response relationship was only partially affected, unlike responses to Iso < 100 nM, which were almost fully inhibited. Fitting of those data to a biphasic concentration-response model yielded EC_50_ values of 8.6 nM and 4.5 μM for the high and low potency phases, respectively, close to the EC_50_ values of 6.3 nM and 2.5 μM measured in the same cells responding to Iso in the presence of extracellular Ca^2+^. Moreover, the amplitude of the Iso-induced maximal responses under nominally Ca^2+^-free conditions (ΔF/F_0_ = 1.1 and 1.3 for the high and low potency phases, respectively) was lower than that determined in the same cells in the presence of extacellular Ca^2+^ (ΔF/F_0_ = 1.5 and 1.4). Thus, under near-zero Ca^2+^ concentration both the Iso response of untransduced cells and the high potency phase of the response in α_1A_-AR transduced cells were largely absent. This finding suggests that the observed β_2_-AR dependent Ca^2+^ mobilization requires an influx of Ca^2+^ from the extracellular compartment, likely via activation of cAMP nucleotide gated-channels. As described in the previous section, the time course of intracellular Ca^2+^ mobilization in response to 100 μM Iso (a concentration that produces high occupancy of α_1A_-ARs) was very similar to that observed for β_2_-AR response and dramatically different from typical Gα_q_-initiated signaling canonical for α_1A_-AR. Remarkably, Ca^2+^ mobilization in response to Iso occupancy of both β_2_-AR and α_1A_-AR was found to be less sensitive to removal of extracellular Ca^2+^ (**Fig. 3B**), indicating intracellular stores as the source of Ca^2+^.

### Iso induced Ca^2+^ mobilization in α_1A_-AR transduced HEK-293/EBNA cells does not reflect coupling to Gα_i_

**Figure 4:**
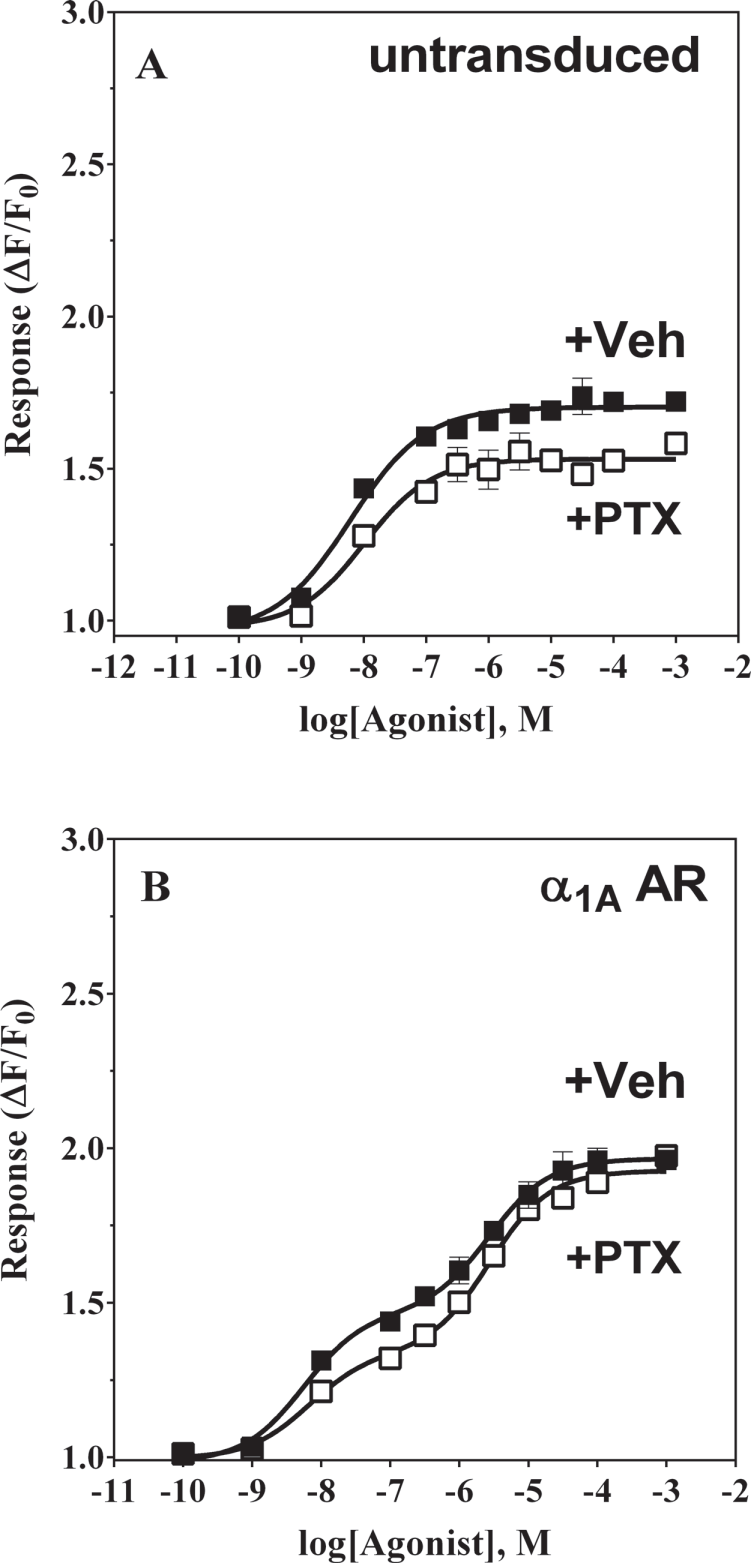
Pertussis toxin pretreatment does not impair Ca^2+^ responses to Iso. HEK-293/EBNA cells were exposed for 3 h to a baculoviral strain carrying α_1A_-AR or to viral medium. Cells were treated with 100 ng/mL of pertussis toxin (PTX) or vehicle for 18 h prior to experiments. **Panel A**: untransduced HEK-293/EBNA cells exposed to PTX (filled symbols) or vehicle (open symbols) were treated with Iso (■,□) during fluorometric imaging of the Ca^2+^-tracking dye. **Panel B:** untreated (open symbols) or PTX-pretreated (filled symbols) α_1A_-AR HEK-293/EBNA cells were stimulated with Iso (■,□) during fluorometric imaging of the Ca^2+^-tracking dye. Each experiment was performed in duplicate two independent times.

Receptor coupling to Gα_i_, the most abundant G protein α subunit, has been shown to lead to activation of PLC activity via the Gβ/γ subunits and thus, can lead to IP accumulation and release of intracellular Ca^2+^ (Reviewed in [36]. Since this pathway is different from Gα_q_-mediated signaling, the kinetics of Ca^2+^ mobilization may also differ. We next searched for a role for Gα_i_ activation in response to Iso-treatment in untransduced and α_1A_-AR transduced cells, pretreated for 18 hours with pertussis toxin or vehicle. Pretreatment of untransduced cells with pertussis toxin (100 ng/mL) resulted in a slight increase in the maximum amplitude of the Ca^2+^ transient response at saturating concentrations of Iso (ΔF/F_0_ = 1.5 vs. 1.7), with a negligible increase in EC_50_ from 6 to 10 nM (**Fig. 4A**)_._ A similar increase was observed in the β_2_-AR–dependent phase of the Iso concentration-response curve (from ΔF/F_0_ = 1.3 to 1.5) in α_1A_-AR expressing cells, also with no change in observed potency (EC_50_ = 6.6 vs. 5.6 nM) (**Fig. 4B**). Since in these cells induction of cAMP leads to Ca^2+^ mobilization [27], increase in Ca^2+^ mobilization following PTX pretreatment may be a result of an increase in intracellular cAMP. This observation would be consistent with previous findings indicating that β_2_-AR can couple to both Gα_s_ and Gα_i_ in HEK-293 [37]. On the other hand, in α_1A_-AR transduced HEK-293/EBNA cells, pertussis toxin treatment did not significantly affect Ca^2+^ mobilization responses to Iso (EC_50_ = 2.8 vs. 2.7 μM with PTX and ΔF/F_0_ = 1.6 vs. 1.5 with PTX), suggesting that coupling to Gα_i_ is not necessary for this Iso-mediated function of α_1A_-AR (**Fig. 4B**).

### Iso does not stimulate formation of inositol phosphates (IP_1,_ IP_2_ or IP_3_) nor IP accumulation in HEK-293/EBNA_α_1A_ AR cells

**Figure 5:**
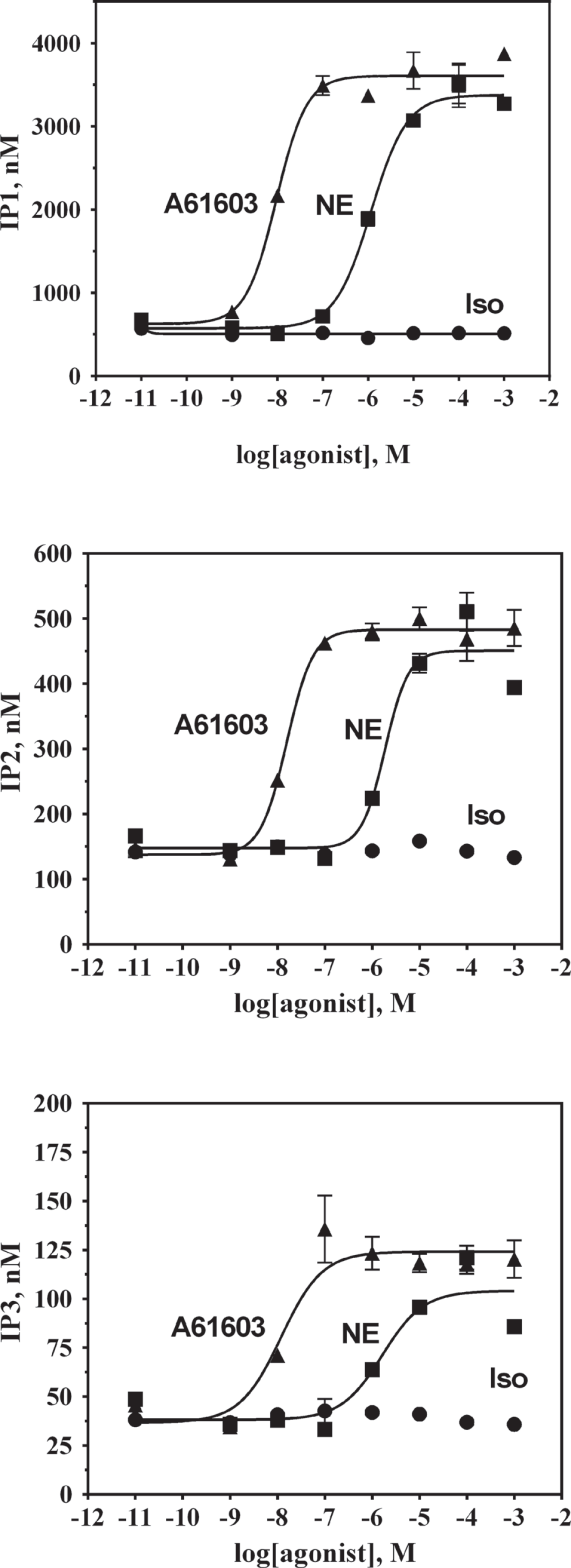
Inositol phosphates production in α_1A_-AR transduced HEK-293/EBNA cells occurs in response to A-61603 and NE, but not in response to Iso. HEK-293/EBNA cells were exposed to baculovirus encoding α_1A_-AR for 3–4 h, then cultured in fresh medium containing 4 mM NaBu for 18 h prior to use in experiments as described in Materials and Methods. IP_1_ (top), IP_2_ (middle) and IP_3_ (bottom) formation was measured in α_1A_-AR transduced HEK-293/EBNA cells stimulated with increasing concentrations of A-61603 (▲), NE (■) or Iso(●). IP_1_, IP_2_, and IP_3_ levels were determined via LC-MS. Plots are representative of three independent experiments with each data point being the average of triplicates.

Given that the α_1A_-AR-dependent phase of the Iso concentration-response relationship was found to be only partially dependent on the extracellular Ca^2+^ and insensitive to pertussis toxin treatment, we asked whether Iso-occupancy at α_1A_-AR results in Gα_q_ activation. In α_1A_-AR transduced HEK-293/EBNA cells, the selective α_1A_-AR agonist A-61603 elicited concentration-dependent IP accumulation (**S1 Fig.**). A-61603-stimulated IP accumulation was best described by a single phase sigmoidal equation yielding an EC_50_ of 40 nM (**Table 1**). Furthermore, preincubation of those cells for 5 minutes with 10 nM of the α_1A_-AR selective antagonist RS100329 significantly attenuated IP accumulation and produced a large, rightward shift in A-61603 potency. In contrast, when replicate cells were treated with Iso (up to 10 mM) no significant accumulation of IP was detected. Furthermore, IP formation in untransduced cells upon treatment with either A-61603 or Iso was not detectable (data not shown). We employed an LC-MS method to increase our detection sensitivity and to measure individual inositol phosphates rather than total IPx accumulation (**Fig. 5**). In α_1A_-AR transduced HEK-293/EBNA both NE and the selective α_1A_-AR agonist A-61603, produced concentration-dependent increases in cellular levels of inositol phosphates IP1, IP2 and IP3 that were best described by a monophasic sigmoidal equation (**Fig. 5**). On the other hand, when replicate cells were treated with Iso (up to 10 mM), no significant changes in intracellular inositol phosphates were detected. Thus, it appears that Iso occupancy at α_1A_-AR biases receptor signaling toward a Gα_q_-independent pathway.

### Iso induces biphasic concentration-response behavior for p-ERK formation in α_1A_- AR transduced HEK-293/EBNA cells

**Figure 6:**
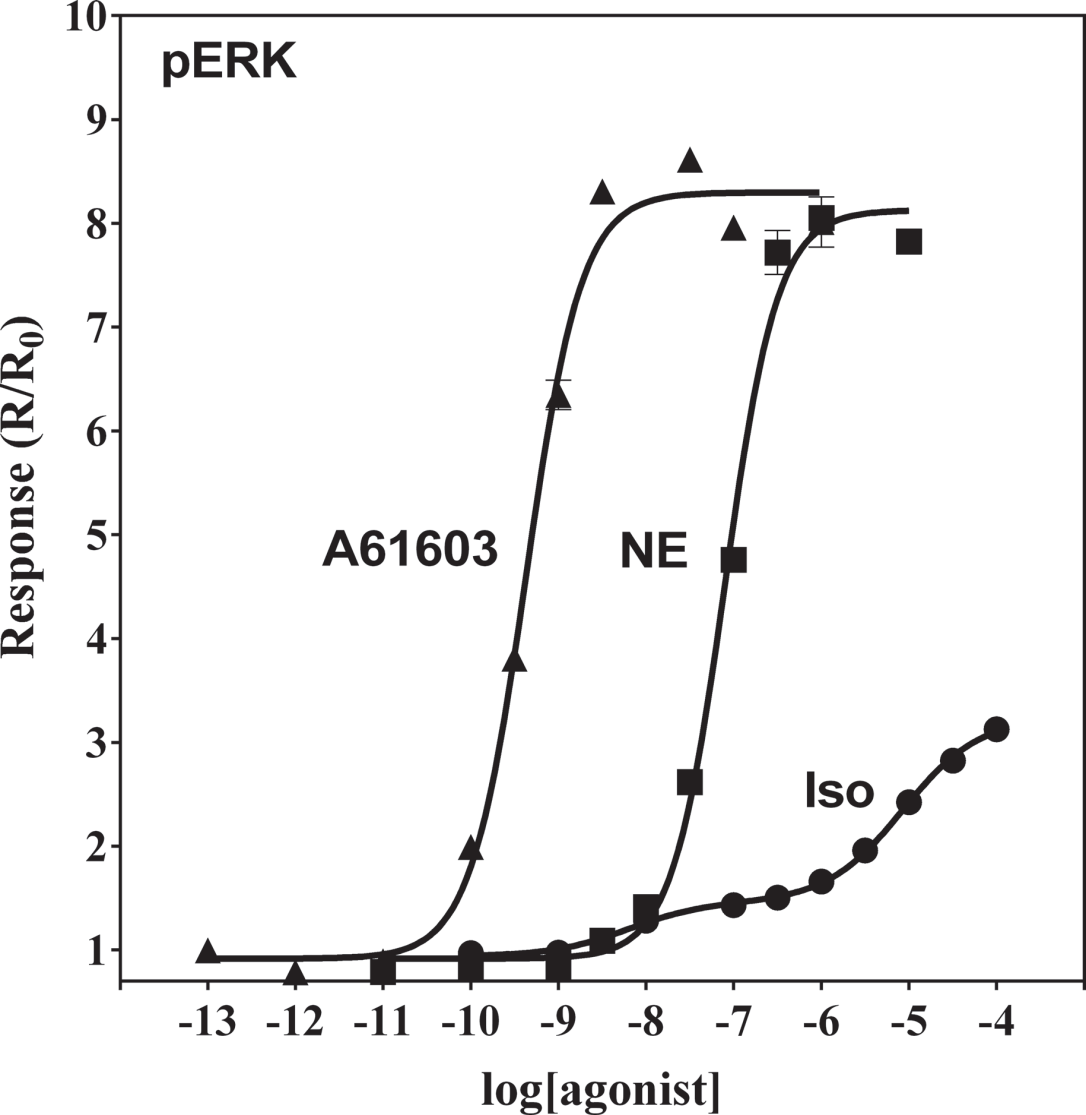
MAPK activation in α_1A_-AR transduced HEK-293/EBNA cells treated with A-61603, NE or Iso. α_1A_-AR transduced HEK-293/EBNA cells were pre-treated with NaBu for 18 h to induce receptor expression. Cells were stimulated with A-61603 (▲), NE (■) or Iso(●) for 5 min. Agonist treatment was terminated by addition of of SureFire lysis solution. Samples were analyzed for levels of phospho-ERK using an AlphaScreen *SureFire* p-ERK assay kit. Plots are representative of four independent experiments, with each data point being the average of triplicates.

Significant efforts have been placed in understanding the role of the mitogen-activated protein kinase (MAPK) cascade in adrenoceptor signaling (*e.g.* in cardiomyocyte physiology, see [38]). We decided to test whether the Iso-mediated signaling that we observed involved this signaling pathway. For this, we examined whether Iso induces ERK1/2 activation. Treatment of α_1A_-AR transduced HEK-293/EBNA cells with Iso induced a dose-dependent increase of p-ERK formation (**Fig. 6**, filled circles). The resulting concentration-response relationship was best fit by a biphasic model. The calculated EC_50_ values were 5 nM and 6 μM for the high and low potency phases, respectively. In untransduced cells a monophasic relationship was observed (EC_50_ = 1 nM, **S2 Fig.**). Interestingly, in α_1A_-AR transduced HEK-293/EBNA cells, A-61603 or NE yielded concentration-dependent activation of ERK that was best fit by a monophasic sigmoidal model. The measured EC50 values were 0.8 and 90 nM for A-61603 and NE, respectively (**Fig. 6**, filled triangles or squares, and **Table 1**). In those cells, A-61603 appeared to be almost 4 times more potent at inducing p-ERK formation than in effecting Ca^2+^ mobilization (EC_50_ = 3 nM, **Table 1**), while NE had about the same potency for both response modes.

Iso stimulation of α_1A_-AR _ HEK-293/EBNA cells resulted in a biphasic concentration-response relationship for both p-ERK formation and Ca^2+^ mobilization transients. The high potency phase of response to Iso (via occupancy of β_2_-AR) and the low potency phase (via occupancy of both β_2_-AR and α_1A_-AR) were quite similar for the two different readouts (**Table 1**).

### Stimulation of α_1A_-AR transduced HEK-293/EBNA with A-61603 and Iso leads to an increase in the level of intracellular α_1A_ – AR without recruitment of arrestins

**Figure 7:**
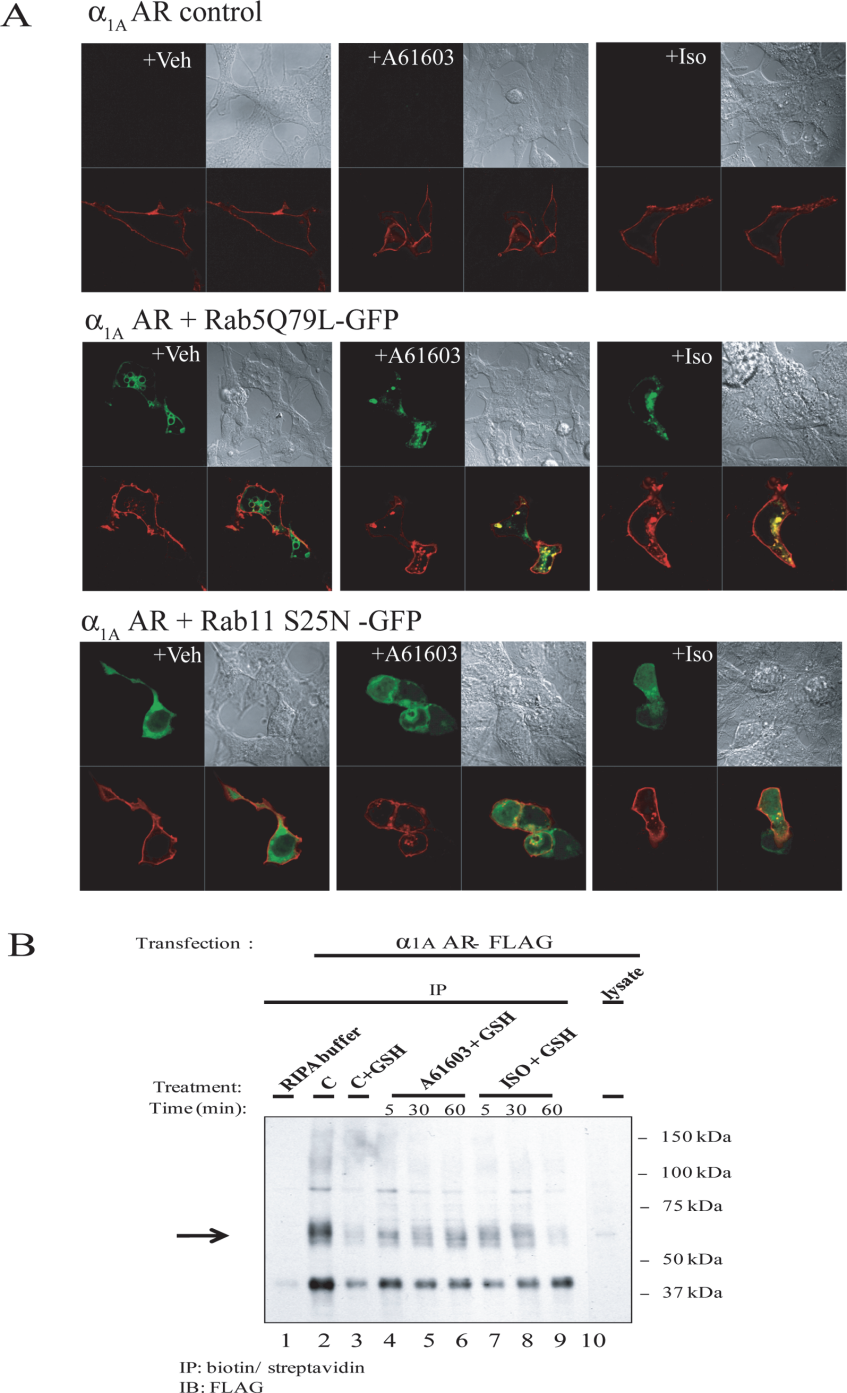
Stimulation of α_1A_-AR transduced HEK-293/EBNA with A-61603 and Iso leads to an increase in intracellular α_1A_ – AR. **A**. HEK293 cells were transiently transfected with α_1A_ – AR only (top panels), or co-transfected with α_1A_ – AR and Rab5 variant Q79L (middle panels) or Rab11 variant S25N (bottom panels). Following serum-deprivation, cells were stimulated with vehicle, 1μM A-61603 or 1mM ISO for 2h. Cells were then fixed and analyzed by confocal microscopy. **B**. HEK293 cells were transiently transfected with α_1A_ – AR. After serum deprivation for 24h, cells were pre-treated with a membrane impermeable, disulfide-cleavable biotin reagent to label plasma membrane α_1A_ – AR. Cells were then left untreated, or stimulated 1 μM A-61603 or 1mM ISO for 5, 30, or 60 min. After treatment, one dish of control cells was harvested without any further manipulations (C: total α_1A_ – AR). The remaining seven dishes were divided into one control (C+GSH), three treated with A-61603 (A-61603+GSH) and three treated with ISO (ISO+GSH). They were stripped of surface biotin label using a reducing agent, in order to reveal internalized, labeled α_1A_ – AR. Samples were then analyzed by immunoprecipitation (IP) with streptavidin followed by immunoblotting (IB) with an anti-FLAG antibody.

**Figure 8:**
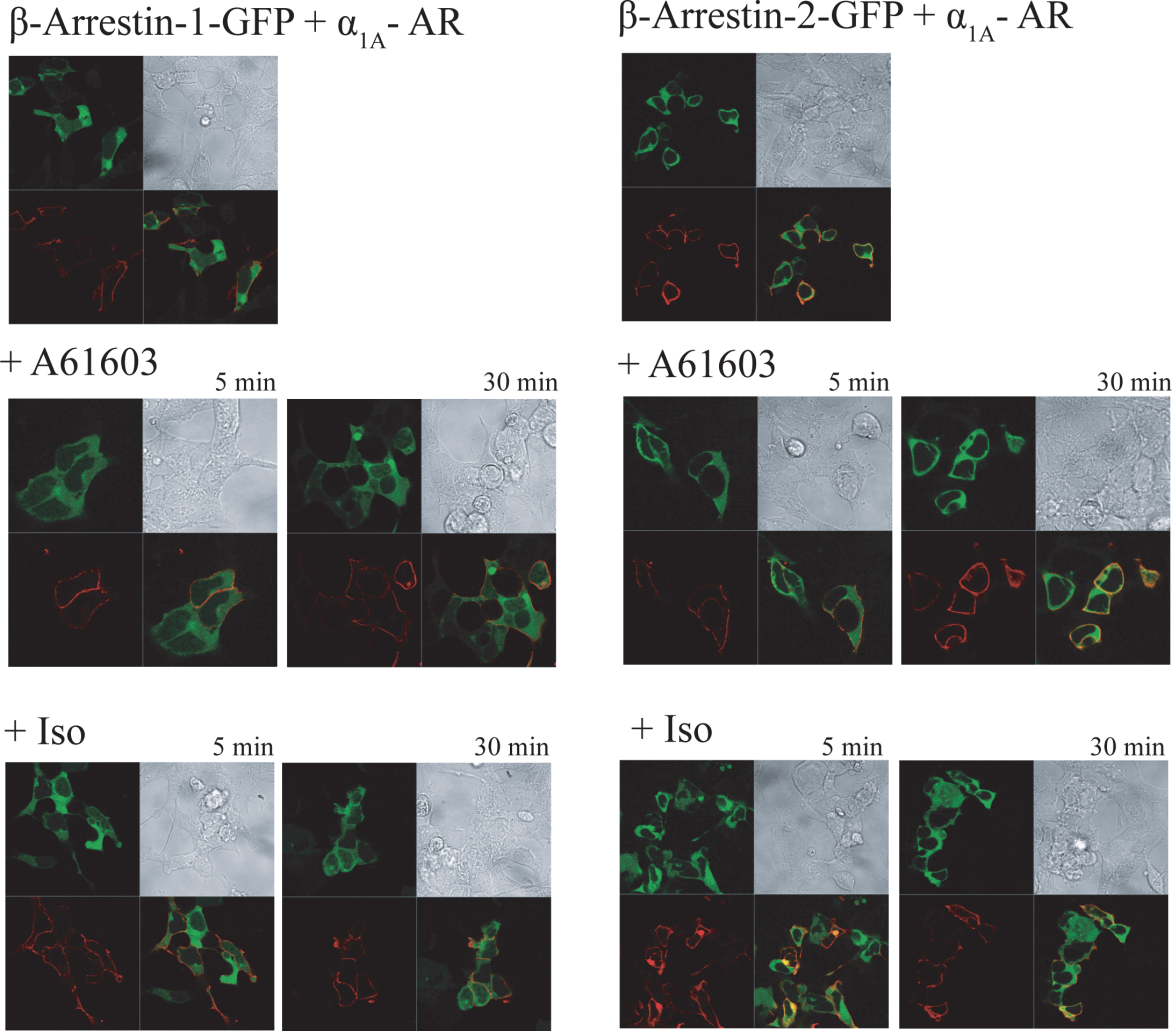
Treatment of α_1A_-AR transduced HEK-293/EBNA with A-61603 and Iso does not trigger intracellular redistribution of arrestins. HEK293 cells were co-transfected with FLAG-tagged α_1A_ – AR and GFP- tagged β-arrestin-1 (left panels) or β-arrestin-2 (right panels). Following serum-deprivation for 24h, cells were left untreated (top panels), or stimulated with 1μM A-61603 (middle panels) or 1mM Iso (bottom panels) for the indicated amount of time. Cells were then fixed, permeabilized, stained with Alexa Fluor-568 conjugated anti-FLAG antibodies, and analyzed employing confocal microscopy.

We next investigated whether or not α_1A_– AR internalizes upon stimulation with A-61603 or ISO. In several prior studies α_1A_– AR has been found to undergo constitutive, ligand independent trafficking and to internalize rather modestly (∼20%) upon agonist stimulation [39–41]. Furthermore this receptor cycling involves clathrin-coated vesicles and entry to early endosomes [39]. To eliminate some of the experimental challenges and uncertainty related to detection of the modest and transient internalization of α_1A_– AR, we employed constitutively active and dominant-negative forms of Rab5 and Rab11, respectively, to the analysis of α_1A_– AR endocytosis using confocal microscopy. Constitutively active Rab5 Q79L enhances endocytosis and early endosome fusion, causing the formation of enlarged early endosomes, and its overexpression has been shown to inhibit transferrin recycling. Rab11 S25N is defective in GTP binding and impairs recycling by inhibiting exit from sorting endosomes (early endosomes) to the recycling endosomes and/or plasma membrane. In HEK-293/EBNA cells transfected with α_1A_ – AR only and stimulated with A-61603 or ISO, α_1A_ – AR was mostly localized at the plasma membrane, as was the case for the vehicle control (**Fig. 7A**). Overexpression of Rab5Q79L caused some intracellular α_1A_ – AR accumulation in vehicle control cells, indicative of constitutive trafficking, as well as significant α_1A_ – AR accumulation upon A-61603 or ISO treatment. Overexpression of Rab11 S25N led to some accumulation in A-61603 or ISO-treated cells, not as pronounced as the one observed upon Rab5Q79L overexpression. In vehicle-treated cells, α_1A_ – AR appeared predominantly at the plasma membrane. To confirm the observation that stimulation with Iso led to internalization of α_1A_ – AR, we took advantage of a cleavable biotin labeling reagent to discern levels of surface versus internalized α_1A_ – AR by means of immunoprecipitation (**Fig. 7B** and **S3 Fig.**). Intracellular α_1A_ – AR could be detected at low levels in control cells (lane 3, “C+GSH”) indicative of constitutive trafficking. Treatment with A-61603 caused an increase in intracellular α_1A_ – AR levels after 5, 30, and 60 min of treatment (lanes 4–6, “A-61603+GSH”) relative to control cells (lane 3,“C+GSH”). Similarly, treatment with ISO caused an increase in the intracellular α_1A_ – AR (lanes 7–8, “ISO+GSH 5 or 30”), as compared to control cells (lane 3, “C+GSH”). We next examined whether Iso-induced internalization of α_1A_ – AR in HEK-293 cells involved the recruitment of β-arrestins. The ability of the N-terminally FLAG-tagged α_1A_-ARs to trigger the translocation of GFP-tagged β-arrestins in those cells was investigated by confocal microscopy. As shown in **Fig. 8**, in vehicle treated cells, the α_1A_-AR was localized mainly at the plasma membrane while both β-arrestins 1 (left panels) and 2 (right panels) were homogenously distributed throughout the cytoplasmic compartment with no visible co-localization with the surface receptor. In cells expressing the α_1A_-AR, stimulation with A-61603 for 30 min did not induce significant translocation of either β-arrestin-1 nor β-arrestin-2, to the plasma membrane. Similarly exposure of the same cells to Iso did not cause visible β-arrestin rearrangement. Internalization of the α_1A_-AR was not detected with either agonist by this method.

### Isoproterenol-induced Ca^2+^ mobilization in α_1A_-AR_CHO cells is not accompanied by detectable increases in intracellular inositol phosphates, yet correlates with activation of the MAPK kinase pathway

**Figure 9:**
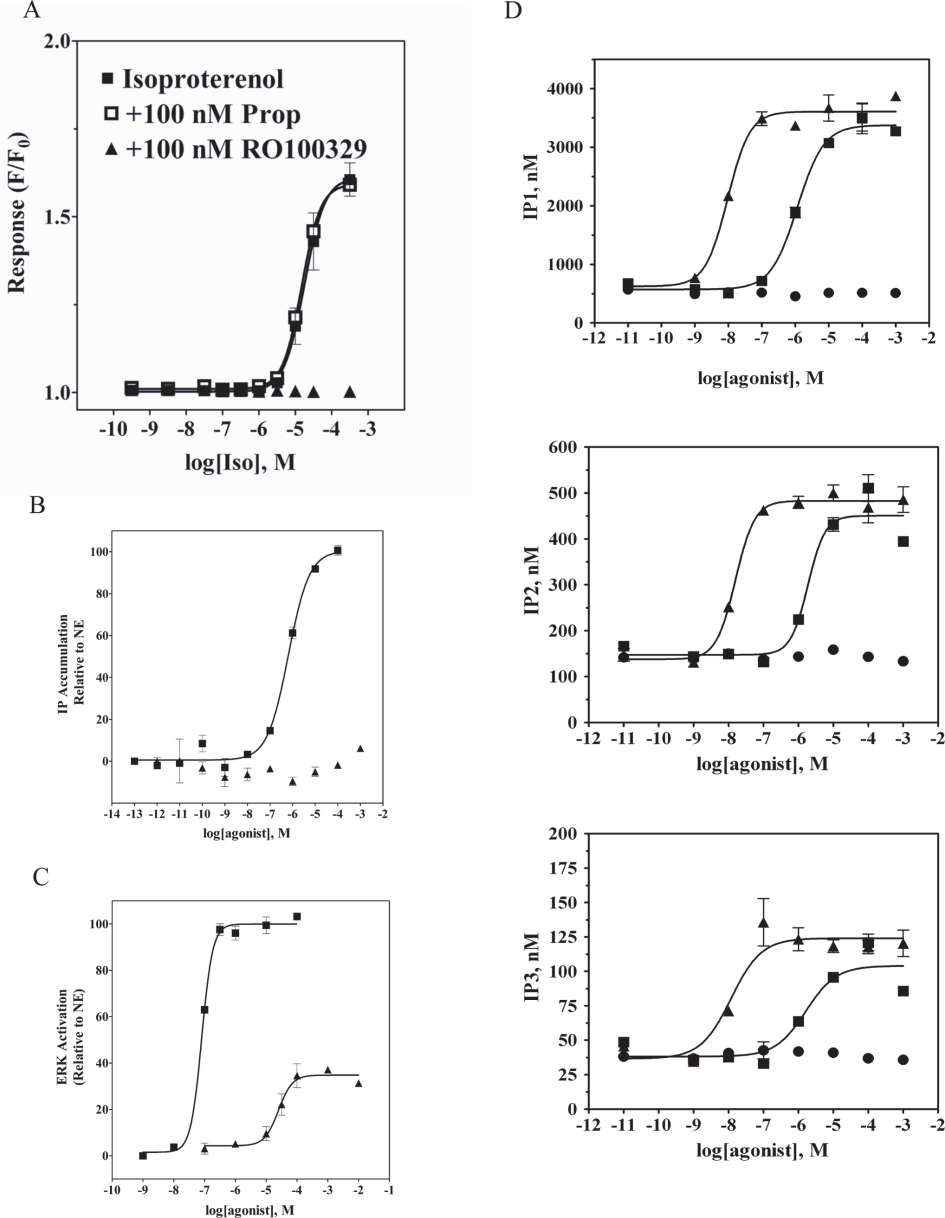
Concentration-response behavior to Iso in Chinese hamster ovary cells stably expressing recombinant α_1A_-AR. **A**. Ca^2+^ transients (expressed as ΔF/F_0_) were measured as a function of Iso concentration in fluo3-loaded cells by fluorometric plate imaging (FLIPR). Responses to Iso were monitored following pre-treatment with either vehicle (■), 100 nM propranolol (□) or 100 nM RO100329 (▲). Inositol phosphate accumulation (**B**) and levels of phospho-ERK (**C)** were measured as a function of increasing concentrations of A-61603 (■) or Iso (▲) and are represented relative to NE. **D**. IP_1_ (top), IP_2_ (middle) and IP_3_ (bottom) formation was measured in CHO cells stably expressing α_1A_-AR, stimulated with increasing concentrations of A-61603 (▲), NE (■) or Iso(●). IP_1_, IP_2_, and IP_3_ levels were determined via LC-MS. Plots are representative of three independent experiments with each data point being the average of triplicates.

Since in HEK-293 cells Iso can stimulate both endogenous β_2_-AR as well as transduced α_1A_-AR, we next tested the activity of this β–agonist at α_1A_-AR in CHO cells stably expressing moderate levels of the receptor (∼1 pmol/mg of membrane proteins) [42]. The β-AR selective agonist Iso evoked a Ca^2+^ transient response in α_1A_-AR_CHO cells (**Fig. 9A)** that was not observed in CCR5_CHO cells used as a negative control (**S4 Fig.**). The peak amplitude of Ca^2+^ transient response, ΔF/F_0_, as a function of agonist concentration yielded a monophasic concentration-response curve with the half-maximal amplitude occurring at 20 μM Iso (**Fig. 9A**). That response to Iso was blocked by preatreatment with 100 nM RO100329, but was insensitive to propranolol at 100 nM. We next examined whether Iso-mediated Ca^2+^ mobilization involved formation of inositol phosphates. Stimulation of α_1A_-AR_CHO cells with NE led to concentration-dependent IP accumulation with an EC_50_ of 0.6 μM (**Fig. 9B**). On the other hand, no IP accumulation could be detected in the same cells stimulated with Iso at concentrations up to 1 mM. Similarly, in α_1A_-AR CHO cells both NE, and the selective α_1A_-AR agonist A-61603, produced concentration-dependent increases in cellular concentration of inositol phosphates; IP1, IP2 and IP3 (**Fig. 9D**) that were best described by a single-site sigmoidal equation. In contrast, when replicate cells were treated with Iso (up to 1 mM) no significant changes in intracellular levels of inositol phosphates could be detected. Thus, it appears that Iso occupancy at α_1A_-AR biases receptor signaling to a Gα_q_-independent pathway. Yet, similarly to the observations made with α_1A_-AR_HEK-293/EBNA cells, stimulation of α_1A_-AR_CHO cells with Iso resulted in a concentration dependent increase in phospho-ERK formation (**Fig. 9C**). The amount of p-ERK formed at the maximal concentration of Iso represented 35% of the level observed in these cells when stimulated with saturating concentrations of NE or A-61603. Iso was less potent than NE and A61603 at inducing ERK activation in α_1A_-AR_CHO cells (Iso: EC_50_ = 17±5 μM; NE: EC_50_ = 0.13±0.02 μM; A61603: EC_50_ = 0.8 ± 0.2 nM). These results indicate that Iso is ineffective at inducing α_1A_-AR-mediated Gα_q_ coupling and PLC activation, yet shows partial agonist activity at mediating α_1A_-AR induced activation of the MAPK signaling cascade.

## Discussion

A search on “isoproterenol” in the PubMed database retrieves more than thirty thousand citations. This potent, β-AR-selective agonist has been extensively studied with respect to many aspects of its action at β-ARs. It has been applied frequently as a tool to verify the presence of β-ARs in cell-based systems or to elucidate the mechanism of β-AR signaling in non-recombinant, native cells such as cardiomyocytes, adipocytes and many others, as well as in organ tissue preparations. In several of these studies the concentrations used were in the micromolar range, which may have triggered off-target effects. Dating back to the 1950s, several reports have indicated that Iso at higher concentrations can evoke α-AR-mediated responses. For instance, Furchgott [9] observed that Iso at concentrations 1–500 μM caused contraction of rabbit thoracic aorta, a tissue in which all three α_1_- AR subtypes are expressed (reviewed in [43]. These researchers also noted that the rate of shortening on addition of Iso was much slower relative to epinephrine or norepinephrine, indicating a distinct mechanism of action for Iso. The maximal contractile response to Iso was about 75% of that obtained for norepinephrine indicating that Iso acted as a partial agonist.

Our studies of HEK-293/EBNA cells with transient low expression levels of α_1A_-ARs cells, to mimic tissues that possess both α_1A_ and β-ARs, provide for the first time a mechanism to account for Furchgott’s original observations. In those cells, a biphasic dose-response relationship was found for Iso-evoked calcium transients. The high potency phase of the response was blocked by non-selective as well as β_2_-selective antagonists, while the low potency phase was sensitive only to α_1A_-AR-selective antagonists. Remarkably, Ca^2+^ transient responses to Iso mediated by α_1A_- ARs exhibited a time course distinctly slower than those seen with prototypical α_1A_ AR agonists (NE, A-61603) that are highly efficacious for IP_x_ formation responses and considered typical for Gα_q_-initiated signaling. Responses to Iso showed delayed onset by 5 to 10 seconds and were much slower to rise in amplitude. Moreover, Iso was a partial agonist at α_1A_- ARs as recorded by this readout. Finally, the observed Ca^2+^ transients exhibited almost complete desensitization, unlike responses to NE. These data taken together indicate a mechanism of action distinct from classical Gα_q_ coupling for Iso at the α_1A_-AR, and suggest functional selectivity (effector signaling bias) for the activity of this agonist.

Agonist occupancy of any given GPCR may trigger signaling events through activation of more than one physiological response cascade. For instance, α_1A_-AR is widely known to couple to phospholipase C. However, this receptor also reportedly mediates activation of several other effectors such as phospholipase D, phospholipase A_2_, adenyl cyclase and several members of the MAPK family (reviewed in [44]). Certain synthetic agonists have been found to be capable of biasing GPCR signaling toward a particular proximal effector. This ability of a ligand to direct an activated receptor state to a particular effector output has been termed “functional selectivity” or “effector bias” (for reviews see [45–48]. Within the adrenoceptor family, there is both pharmacological and biophysical evidence for agonist dependent effector bias at β-ARs. Signaling bias has been shown for several ligands at both β_1_- and β_2_-AR toward adenyl cyclase and MAPK [49,50].

We observed that Iso occupancy of α_1A_-AR caused Ca^2+^ transient responses in HEK-293/EBNA cells without observable PLC activation: no formation of individual inositol phosphates or IP accumulation was detected in these cells under the same conditions. Unexpectedly, Iso occupancy of α_1A_-AR also mediated ERK1/2 activation, suggesting that Gα_q_ coupling is not required for ERK activation by Iso. Adding to the surprise, A-61603 appeared to be significantly more potent at activating ERK as compared to activation of Gα_q_, whereas NE displayed equivalent potencies for both responses. Taken together, the signaling patterns observed with these agonists appear to reveal a spectrum of partially overlapping effector coupling mechanisms. Iso, and the imidazoline A-61603, appear to have a bias toward MAPK activation. Moreover, for A-61603 there seems to be greater receptor reserve or response amplification for the pERK formation response.

The α_1A_-AR has also been found to regulate mitogenic responses, but it is not clear if this activity is downstream of Gα_q_ engagement [38,51]. Minneman and co-workers have in fact reported NE activation of α_1A_-AR-mediated MAPK phosphorylation in rat PC12 cells [51,52], independently of both Gα_q_-induced Ca^2+^ mobilization and PKC activity [53]. The same group later showed that deletion of the first three amino acids from the third intracellular loop not only uncouples the receptor from Gα_q_, but also abolishes activation of the MAPK pathway [54]. It was suggested that these two pathways operate independently from one another, yet may depend on similar structural elements of agonist-occupied receptor. Several lines of evidence seem to indicate that in mouse cardiomyocytes, the α_1A_-AR-mediated MAPK activation may not be associated with activation of Gα_q_ pathway [38,55]. α_1A_-AR /α_1B_-AR double-knockout mice also developed heart failure after transverse aortic constriction, and reconstitution of α_1A_-AR signaling in cardiomyocytes from those animals rescued them from NE-induced apoptosis [56]. The expected IP generation was not detectable in those cardiomyocytes with reconstituted α_1A_-AR function or in wild-type mouse cardiomyocytes, pointing at a non-Gq signaling mode for this receptor in myocardial function. More importantly, Huang et al. (2007) reported that α_1A_-AR stimulation of an ERK-mediated pathway is critical for cardiomyocyte survival. Our data support those findings as well as provide further evidence indicating that Iso occupancy of α_1A_-AR induces an active receptor “state” that leads to MAPK pathway activation without activating the canonical Gα_q_ pathway.

Our previous studies of Gα_q_ coupling defective variants of α_1A_-AR combined with examination of the effects of Ca(II) channel blockers uncovered cross-talk between α_1A_-AR and β_2_-AR that leads to potentiation of a Gα_q_-independent signaling cascade in response to α_1A_-AR activation [27]. Moreover, this signaling event was accompanied by Ca(II) mobilization with unusual kinetics mirroring the kinetics observed for Iso induced Ca(II) mobilization in α_1A_-AR_HEK293/EBNA cells. Interestingly, in the presence of a β-AR-selective antagonist, the amplitude of the α_1A_-AR-mediated Ca^2+^ transient response to Iso (*i.e.* the lower potency phase of the Iso concentration-effect relationship) was not only lower than expected, but also occurred with a significant delay in onset. This may indicate a synergy occurs between these receptors in generating the lower potency phase of response. Depletion of extracellular Ca(II) did not affect Iso induced activation of α_1A_-AR indicating that intracellular stores were the source of Ca(II) released. Finally, this signaling cascade did not involve coupling to Gα_q_, Gα_s_ or Gα_i_ but led to receptor internalization and activation of ERK1/2. An alternate mechanism for the control of Ca^2+^ release from the ER involves ryanodine receptors (RyRs). They have been shown to exist in several non-excitable cell lines, although their functional expression in HEK-293 cells is controversial. RT-PCR analysis of HEK-293/EBNA cells found that significant levels of RyR2 mRNA are present in these cells.

Recent reports provide an independent line of evidence for α_1A_-AR-mediated activation of endocytic pathway that leads to phosphorylation of ERK1/2, independent of Gα_q_ /PLC/PKC signaling [57,58]. In one study, dynamin mutants were employed to induce trafficking defects, along with various molecules disrupting actin and tubulin organization. Liu and coworkers show that α_1A_-AR induced activation of ERK1/2, but not p38 MAPK, was dependent on cytoskeleton and actin organization. On the other hand, α_1A_-AR-induced activation of PKC and C-Raf was not affected by endocytosis disruption. Neither PKC nor PLC inhibition blocked α_1A_-AR induced activation of ERK1/2 [57]. More recently, the spatial-temporal characteristics of receptor internalization and ERK1/2 activation in response to stimulation of α_1A_-AR in HEK293 were investigated in combination with various inhibitors of PKC, receptor internalization as well as β-arrestin 2 silencing and differential patterns were discovered for Gq/PKC dependent as compared to the Gα_q_-independent signaling cascade. The rapid phosphorylation of ERK1/2 was dependent on activation of PKC downstream of Gα_q_ and resulted in pERK1/2 translocation into the nucleus while sustained activation of ERK1/2 that was limited to cytoplasmic compartment, was independent of PKC but dependent on receptor internalization into acidified endosomes and was mediated by β-arrestin 2[58]. Together, those findings and our own data provide strong evidence that α_1A_-AR can induce activation of ERK1/2 in a manner that is independent of the canonical Gα_q_ /PLC/PKC signaling cascade but involves receptor internalization. We also found that Iso is a biased agonist that can preferentially stimulate α_1A_-AR mediated activation of MAPK signaling pathway through receptor internalization and without induction of Gα_q_ coupling. Although, our study did not conclusively detect Iso induced α_1A_-AR association with β-arrestins after agonist application, involvement of β-arrestin 2 in mediating this response cannot be ruled out. Several groups have demonstrated that agonist-mediated GPCR association with β-arrestin leads to G protein-independent activation of the MAPK pathway [23]. In fact, it has been shown recently that NE occupied α_1A_-AR associates although weakly with both β-arrestin-1 and 2, but coupling to the MAPK pathway was not investigated in this study [59]. Further studies including β-arrestin knock-downs will be needed to fully determine whether or not this signaling mechanism is actually dependent on β-arrestin 2.

On the other hand, it has been recently shown that a genetic variant of the α_1A_-AR transactivates EGFR via a β-arrestin1-dependent mechanism similarly to the β1-AR mediated EGFR transactivation and leads to ERK1/2 activation downstream of EGFR [60–62]. Transactivation of EGFR by β-AR confers cardioprotection [61,62] suggesting that transactivation could be involved in the antiapoptotic and cardioprotective activity of the α_1A_-subtype. We have examined this potential mechanism in mediating the Iso induced α_1A_-AR stimulation of ERK1/2 using EGFR inhibitor AG1478 and found that activation of ERK was not affected by the inhibition of EGFR (data not shown). Further studies may be needed to fully explore this mechanism.

Our observations differ from those of Sun *et al*., who reported that Iso evoked biphasic dose-effect relations in MAPK activation [24]. The observed biphasic behavior was attributed to distinct processes by which MAPK activity is modulated, either through regulation of adenylyl cyclase by β_2_-AR/Gα_s_, or by formation of a macromolecular complex containing β_2_-AR and c-Src which directly activates c-Src. The conclusion that β_2_-AR was the sole mediator of both phases of the Iso response was based on their complete blockade by 1 μM ICI118551, as well as lack of detectable MAPK activation in MEF cells derived from β_1_-AR^−/−^ and β_2_-AR^−/−^ mice. Although these findings offer strong evidence for β_2_-AR involvement in mediating that response, they do not necessarily rule out a synergy between β_2_-AR and α_1_-ARs, if present in those cells.

Interestingly, we observed that in the presence of a β-AR-selective antagonist, the kinetics of Iso-evoked α_1A_-AR-mediated Ca(II) transient response was much slower and with the greatest delay (**Fig. 2C**), and the the amplitude of the α_1A_-AR-mediated Ca(II) transient (*i.e.* the lower potency phase of the Iso concentration-effect relationship- **Fig. 1B**) was lower than expected, indicating synergy between these receptors in generating the low potency response phase. This is consistent with previously published results with wild-type α_1A_ AR and Gαq signaling deficient variants expressed in HEK EBNA cells with endogenous expression of β2 AR that showed that β2 AR signaling actually potentiates α_1A_ AR- mediated Ca(II) response and the potentiation was most striking in cells expressing Gαq defective mutants [27]. Furthermore, this potentiation of Ca(II) transient was most evident for the signal not blocked by Xestospongin C/2-APB application, and consistent with the non-Gq /ERK signaling cascade.

Nonetheless, Iso can also induce intracellular Ca^2+^ mobilization (as well as ERK activation) in CHO cells lacking β_2_-AR expression, upon stable expression of α_1A_-ARs (**Fig. 9**) and in α_1A_-AR_ HEK-293/EBNA cells in presence of β-AR antagonists indicating that this signaling is not dependent on β_2_-AR. The potency of Iso at mediating the Ca(II) response in α_1A_-AR_ CHO cells (EC_50_ = 18 ± 8 μM) was somewhat lower than the potency of the second phase observed in HEK-293/EBNA cells (EC_50_ = 2.6 μM). This response was blocked by an α_1A_-AR-selective antagonist, but was not sensitive to the β-AR selective antagonist propranolol. On the other hand, in untransduced HEK-293/EBNA cells expressing β_2_-ARs which lack α_1A_-AR expression, we observed only a high potency, monophasic response to Iso for both intracellular Ca^2+^ mobilization and p-ERK formation.

Although, this study utilizes a model system where α_1A_-AR was transiently expressed at low, physiological levels together with endogenous β2-AR in HEK293(EBNA), evidence already exist suggesting that similar mechanism may be utilized in physiologically relevant settings such as *e.g.* cardiomyocytes where both receptors are co-expressed. Sabri *et al* observed that in rat cardiomyocytes ERK activation in response to NE was mostly mediated by α_1_-AR receptor and only a minor component of ERK was activated by β-AR[63]. In the same cardiomyocytes Isoproterenol induced p38-MAPK, cAMP and enhanced contractile function at concentrations that retained β2-AR selectivity while ERK1/2 activation required 100 fold higher dose (of 10 μM). On the other hand Zinterol induced ERK activation at β2-AR selective concentrations indicating that receptor reserve was not the problem. Role of other adrenoceptors in mediating ERK1/2 activation in response to ISO was not addressed. In a more recent publication cardioprotective and cardiotoxic effects of Isoproterenol on feline cardiomyocytes *in vitro* and *in vivo* have been reported. The cardiotoxic effect was found to be mediated by PKA and sarcoplasmic Ca(II) overload downstream of β-AR since the effect could be blocked by PKA inhibitory peptide and β-AR antagonist. On the other hand the cardioprotective effect was attributed to ERK activation mediated by EPAC downstream of β-AR [64]. Although strong evidence implicating EPAC in the Iso mediated cardioprotective signaling was presented, the response was induced by high non-selective Iso concentrations (10 μM) and could not be fully blocked by β-AR antagonist metoprolol (at 20 mg/kg body weight) implicating potentially involvement of other non-β-AR receptor. Interestingly, earlier studies performed with α_1A/B_-AR KO mice reported that α_1A_-AR induced ERK signaling pathway is cardioprotective and required for cardiomycocyte’s survival[38]. The hearts of AB KO mice had worse fibrosis and increased cardiac cell death as compared to those from wild-type littermates when pressure loaded, while NE or Iso treatment of cultured cardiomyocytes from the KO mice resulted in more necrosis and apoptosis. The enhanced susceptibility to cell death could be rescued by reintroduction of α_1A_ –AR but not α_1B_-AR and required activation of ERK1/2. Similarly, in neonatal rat myocytes, α_1_-AR stimulation inhibited apoptosis caused by cAMP, and was abolished by a MEK inhibitor suggesting a role for ERK1/2[65]. Recent evidence points also at potential protective effects of α_1A_ –AR signaling in the CNS (reviewed in [66]). Chronic α1A-AR stimulation was shown to increase neurogenesis, enhance learning and memory while also protecting the brain from anoxia and traumatic injury, seizures, and age-dependent neurodegeneration.

In conclusion, we have found that isoproterenol is a low potency agonist at α_1A_-ARs, and appears to manifest effector bias when bound at α_1A_-AR, inducing activation of a Gα_q_-independent signaling cascade. This may explain decades-old observations of unusual properties reported for this ligand. As discussed earlier, in cardiomyocytes, induction of the MAPK pathway via α_1_-ARs has been shown to protect those cells from apoptosis [38]. In contrast, increased Gα_q_ signaling induced cardiac hypertrophy and loss of β-AR inotropic responsiveness [67], ultimately leading to heart failure [68]. Thus, our discovery of the apparently biased coupling at α_1A_-AR of the agonist ISO, adds pharmacological and chemical support for the potential to identify a novel class of therapeutics for treatment of heart failure [56,69]. Although, isoproterenol itself will not be a drug candidate in this respect, it can serve as benchmark for the search of molecules with such pharmacological profile. Meanwhile, caution should be taken while using isoproterenol in non-recombinant, undefined systems, with proper controls and/or selective antagonists included in those experiments to minimize confounds attributable to the involvement of α-ARs in monitored outcomes.

## Supporting Information

S1 Fig Inositol phosphate accumulation in α_1A_-AR transduced HEK-293/EBNA cells occurs in response to A-61603 but not in response to Iso. Inositol phosphate accumulation responses are shown for A-61603 (squares) or Iso (circles) in α_1A_-AR transduced HEK-293/EBNA cells after pre-treatment with vehicle (filled symbols) or antagonist (empty symbols). HEK-293/EBNA cells were exposed to baculovirus encoding α_1A_-AR for 3–4 hours and then cultured in fresh media containing 4 mM NaBu for 18 hours prior to use in experiments as described in Materials and Methods. Cells were preincubated for 20 min with vehicle or 10 nM of α_1A_-AR antagonist RS-100329 and then exposed to agonist for 10 minutes. IP concentration was determined by homogenous time-resolved immunofluorescence detection method. The plot is representative of two independent experiments with each data point being an average of quadruplicate.
(TIF)

S2 Fig MAPK activation in α_1A_-AR transduced and mock transduced negative control HEK-293/EBNA cells treated with Iso. HEK-293/EBNA cells that were transduced with baculovirus bearing aldehyde oxidase(as negative control vector) (■) or α_1A_-AR (●), were pre-treated with NaBu for 18 h to induce receptor expression. Cells were stimulated for 5 min with increasing concentration of Iso. Agonist treatment was terminated by addition of of SureFire lysis solution. Samples were analyzed for levels of phospho-ERK using an AlphaScreen *SureFire* p-ERK assay kit. Plots are representative of two independent experiments, with each data point being the average of triplicates.
(EPS)

S3 Fig Stimulation of α_1A_-AR transduced HEK-293/EBNA with A-61603 and Iso leads to an increase in intracellular α_1A_ – AR. HEK293 cells were transiently transfected with α_1A_ – AR. After serum deprivation for 24h, cells were pre-treated with a membrane impermeable, disulfide-cleavable biotin reagent to label plasma membrane α_1A_ – AR. Cells were then left untreated, or stimulated 1 μM A-61603(A) or 1mM ISO(I) for 5, 30, or 60 min. After treatment, one dish of control cells was harvested without any further manipulations (C: total α_1A_ – AR). The remaining seven dishes were divided into one control (C+GSH), three treated with A-61603 (A-61603+GSH) and three treated with ISO (ISO+GSH). They were stripped of surface biotin label using a reducing agent, in order to reveal internalized, labeled α_1A_ – AR. Samples were then analyzed by immunoprecipitation (IP) with streptavidin followed by immunoblotting (IB) with an anti-FLAG antibody. Bands were quantified by densiometry, normalized to control. Plots are representative of three independent experiments.
(EPS)

S4 Fig Concentration-response relation to Iso stimulation in α_1A_-AR-free Chinese hamster ovary cells stably expressing recombinant CCR5. Ca^2+^ transients (expressed as ΔF/F_0_) were measured as a function of Iso concentration (■) in fluo3-loaded cells by fluorometric plate imaging (FLIPR). Cells were stimulated with CCR5 agonist MIP1α (∇, positive control) at 1 μM concentration to confirm their responsiveness. Plots are representative of two independent experiments with each data point being the average of triplicates.
(EPS)
